# Head-to-head comparison of anterior nares and nasopharyngeal swabs for SARS-CoV-2 antigen detection in a community drive-through test centre in the UK

**DOI:** 10.1136/bmjresp-2023-001747

**Published:** 2025-03-22

**Authors:** Rachel L Byrne, Ghaith Aljayyoussi, Konstantina Kontogianni, Karina Clerkin, Mathew McIntyre, Jahanara Wardale, Christopher T Williams, Richard Body, Emily R Adams, Margaretha de Vos, Camille Escadafal, Ana I Cubas Atienzar

**Affiliations:** 1 Centre for Drugs and Diagnostics, Liverpool School of Tropical Medicine, Liverpool, UK; 2 Manchester University NHS Foundation Trust, Manchester, UK; 3 Global Access Diagnostics, Bedford, UK; 4 Nuffield Department of Medicine, University of Oxford, Oxford, UK; 5 FIND, Geneva, Switzerland

**Keywords:** COVID-19

## Abstract

**Objective:**

To conduct a head-to-head diagnostic accuracy evaluation of anterior nares (AN) and nasopharyngeal (NP) swabs for SARS-CoV-2 antigen detection using two brands of rapid diagnostic tests (Ag-RDT).

**Methods:**

Two prospective diagnostic evaluations were carried out at different time points and participant cohorts to evaluate the performance of paired AN and NP swabs in two Ag-RDT brands: Sure-Status (PMC, India) and Biocredit (RapiGEN, South Korea). The sensitivity and specificity of AN and NP swabs for each of the index test cohorts were calculated against the reverse transcription quantitative PCR (RT-qPCR) TaqPath COVID-19 (ThermoFisher, UK) using NP swabs as reference standard.

**Results:**

A total of 372 participants were recruited for the Sure-Status cohort and 232 for the Biocredit, of which 119 (32.1%) and 122 (53.7%) were SARS-CoV-2 positive by RT-qPCR, respectively. Sensitivity and specificity of AN swabs were equivalent to those obtained with NP swabs in both cohorts: 83.9% (95% CI 76.0–90.0) and 98.8% (95% CI 96.6–9.8) using NP swabs and 85.6% (95% CI 77.1–91.4) and 99.2% (95% CI 97.1–99.9) with AN swabs for Sure-Status and; 81.2% (95% CI 73.1–87.7) and 99.0% (95% CI 94.7–86.5) with NP swabs and 79.5% (95% CI 71.3–86.3) and 100% (95% CI 96.5–100) with AN swabs for Biocredit. The agreement of the AN and NP swabs was high for both brands with an inter-rater reliability (κ) of 0.918 and 0.833 for Sure-Status and Biocredit, respectively. The overall 50% limits of detection (LoD50) and 95% LoD (LoD95) were 0.9–2.4×10^4^ and 3.0–3.2×10^8^ RNA copies/mL for NP swabs and 0.3–1.1×10^5^ and 0.7–7.9×10^7^ RNA copies/mL for AN swabs, with no significant difference in LoD for any of the swab types or test brands.

**Conclusions:**

The diagnostic accuracy of the two SARS-CoV-2 Ag-RDT brands evaluated in this study was equivalent using AN swabs than NP swabs. However, test line intensity was lower when using AN swabs, which could negatively influence the interpretation of the Ag-RDT results by lay users.

**Trail registration number:**

NCT04408170.

**WHAT IS ALREADY KNOWN ON THIS TOPIC**

- Studies on SARS-CoV-2 reverse transcription PCR (RT-PCR) testing found that anterior nares (AN) swabs were 12%–18% less sensitive than nasopharyngeal (NP) swabs. Studies on antigens rapid diagnostic tests (Ag-RDTs) using paired AN and NP are still very limited.

**WHAT THIS STUDY ADDS**

- We investigated the diagnostic accuracy of two commercially available SARS-CoV-2 Ag-RDTs using paired AN and NP swabs from symptomatic patients attending a drive-through test centre, and the diagnostic accuracy and the limit of detection were comparable in both swab types of both Ag-RDT brands. However, the test line intensity was lower when using AN swabs, which could negatively influence the interpretation of the Ag-RDT results for lay users.

**HOW THIS STUDY MIGHT AFFECT RESEARCH, PRACTICE OR POLICY**

- The equivalent diagnostic accuracy using both swab types is an advantage as AN sampling could enable scaling up antigen testing strategies. Additional studies on Ag-RDTs using AN swabs on self-interpretation by a layperson are needed to ensure that low-intensity test lines are not classified as false negatives.

## Introduction

 To meet the immense diagnostic demand of the COVID-19 pandemic, the development of rapid diagnostic tests for the detection of SARS-CoV-2 antigens (Ag-RDTs) became a priority.^1^ Nasopharyngeal (NP) swabs are considered the standard of care for SARS-CoV-2 detection^2^ and thus the majority of Ag-RDT kits were developed for NP swabs exclusively.^1^ However, the use of anterior nares (nasal) (AN) swabs has been increasing as a less invasive alternative to promote access to testing in the community and facilitate mass testing programmes particularly in the UK.^3^

For Ag-RDTs, studies comparing sensitivity on AN swabs and NP swabs are very limited. There are three reported studies, one meta-analysis reporting pooled data on AN swabs and NP swabs^4^ from 12 commercially available Ag-RDTs and two studies performed a head-to-head comparison on the same Ag-RDT brand, Standard-Q (SD Biosensor, Korea), one study on professionally taken swabs^5^ and another in self-taken swabs.^6^ Sensitivity obtained with AN swabs was comparable (although 3%–5% lower) to NP swab sensitivity, but neither of the swab types fulfilled WHO target product profile (TPP)^7^ standards in either of the two published studies.^5 6^

AN swabs are considered accurate and clinically acceptable alternatives to NP swabs in outpatient settings for SARS-CoV-2 reverse transcription PCR (RT-PCR) testing.^8^ However, an in-depth meta-analysis on SARS-CoV-2 RT-PCR testing found that AN specimens were 12%–18% less sensitive than NP swabs.^9^

The aim of this study was to perform a head-to-head comparison of AN and NP swabs using two WHO approved for Emergency Use Listing (WHO-EUL) SARS-CoV-2 Ag-RDT brands that are marketed for both sample types: Sure-Status COVID-19 Antigen Card Test (Premier Medical, India) and Biocredit COVID-19 Antigen Test (RapiGEN, South Korea), respectively.

This study is of particular interest in the UK as the use of home Ag-RDTs on AN swabs was integral to combatting the spread of COVID-19 during the pandemic,^3^ as on 1 April 2022 free national RT-PCR COVID-19 testing was suspended, with the purchase of Ag-RDTs using AN swabs online or in pharmacies the only approach to access COVID-19 testing in a non-clinical setting.

## Methods

### Clinical evaluation

The Standards for Reporting of Diagnostic Accuracy statement was adopted as a guideline for study design and reporting. This was a prospective evaluation of consecutive participants enrolled at a community National Health Service drive-through COVID-19 test centre located at the Liverpool John Lennon Airport. Two Ag-RDT brands were evaluated: Sure-Status COVID-19 Antigen Card Test (Premier Medical, India) and Biocredit COVID-19 Antigen Test (RapiGEN, South Korea) referred to as Sure-Status and Biocredit thereafter. Swab samples used for the evaluation of Sure-Status were obtained from participants recruited between August and October 2021, and for the evaluation of Biocredit from participants recruited between December 2021 and March 2022. The study progressed until at least 100 Ag-RDT positives using AN swabs in line with WHO’s requirements for evaluation of alternative sample type.^10^

All adults over the age of 18 who attended the drive-through test centre with symptoms of COVID-19 were asked to participate in the study. The symptoms included fever, cough, shortness of breath, tight chest, chest pain, runny nose, sore throat, anosmia, ageusia, headache, vomiting, abdominal pain, diarrhoea, confusion, rash or tiredness. Participants were recruited under the Facilitating Accelerated COVID-19 Diagnostics study using verbal consent.

Swabs were collected by trained healthcare workers following the same process with the NP swab collected first in one nostril and placed in Universal Transport Media (UTM) (Copan Diagnostics, Italy) for the reference RT-qPCR test. This was followed by the collection of two swabs to evaluate the Ag-RDTs, first an NP swab in the other nostril and finally an AN swab in both nostrils following the manufacturer’s instructions for use (IFU). Samples were given a unique identification code and transported within cooler bags to the Liverpool School of Tropical Medicine where samples were processed in category level 3 (CL3) containment laboratory on arrival by trained research technicians.^11^

Sure-Status and Biocredit Ag-RDTs were carried out following their IFU. The protocol for both Ag-RDTs was the same when using AN and NP swabs. Results were read by two operators, blinded to one another, and if a discrepant result occurred, a third operator acted as a tiebreaker. The visual read-out of the Ag-RDT test band was scored on a quantitative scale from 1 (weak positive) to 10 (strong positive). Ag-RDT results were classified as invalid when the control line was absent. Photos were taken of all Ag-RDTs and results were QC against the reported results by the operators performing and interpreting the Ag-RDT results.

RNA was extracted using the QIAamp 96 Virus QIAcube HT kit (Qiagen, Germany) on the QIAcube (Qiagen, Germany) and screened using TaqPath COVID-19 (ThermoFisher, UK) on the QuantStudio 5 thermocycler (ThermoFisher, UK), an internal extraction control was incorporated before the lysis stage, as recommended by the manufacturer. SARS-CoV-2 RT-qPCR result was considered (1) positive if any two of the three SARS-CoV-2 target genes (N gene, ORF1ab and S gene) amplified with cycle threshold (Ct) ≤40, (2) indeterminate if only one SARS-CoV-2 gene amplified and (3) negative if the internal extraction control amplified and the SARS-CoV-2 target genes did not. Samples with invalid RT-qPCR results (no amplification of the internal extraction control) were re-extracted and re-run once. Viral loads in UTM swabs were measured with a 10-fold serial dilution standard curve of quantified specific in vitro-transcribed RNA using five replicates for each standard curve point.^12^,^13^

### Statistical analysis

Sensitivity, specificity, positive predicted value and negative predictive values were calculated with 95% CIs by comparing the Ag-RDT results by swab type to the RT-qPCR, as the reference standard. Subanalyses of diagnostic performance were performed by swab type (AN and NP), Ct-value ranges, onset of symptoms and vaccination status using non-parametric statistics. The level of agreement between AN and NP swabs was determined using Cohen’s kappa (κ).^10^ The correlation between test line intensity and viral loads was measured by Pearson correlation coefficient (r_P_)^14^ and to further analyse Ag-RDT sensitivities, we used logistic regression, with RNA copy numbers of the RT-qPCR NP swab and swab type (AN and NP) as independent variables and test outcomes as the dependent variable, yielding detection probabilities for each viral load level. Statistical analyses were performed using SPSS V.28.0, Epi Info V.3.01 and R scripts. Statistical significance was set at p<0.05.

### Patient and public involvement

Participants were not involved in setting the research question, the outcome measures or the design and implementation of the study. Patients did not receive the results of the RDTs evaluated in the study.

## Results

### Participant demographics

A total of 604 participants were recruited for this study, 372 recruited between August and October 2021 were enrolled for the Sure-Status Ag-RDT evaluation and 232 recruited between December 2021 and March 2022 were enrolled for the Biocredit Ag-RDT evaluation (online supplemental material 1). Details of the demographics of the population of study are found in table 1. Our study population had a mean age of 43 years (range 18–81, IQR 33.0–50.0), 348 (58%) were female and 566 were British (94%), with the remaining 36 participants being of other nationalities. 314 participants of the 372 enrolled for the Sure-Status evaluation (84.4%) and 217 participants of the 232 recruited for Biocredit (93.5%) received complete SARS-CoV-2 vaccination (2 doses). Additionally, 143 of the participants enrolled from December 2021 (61.6%) for the Biocredit evaluation received a third dose as part of the UK booster roll-out.^15^ All participants were symptomatic with a median onset of symptoms of 2 days (IQR 1–3). The most common symptoms were cough (387, 64.3%), sore throat (232, 38.5%), headache (203, 33.7%), fever (160, 26.6%), body aches (80, 13.3%) and runny nose (80, 13.3%) (table 1).

**Table 1:** Demographics of the population of study for Sure-Status and Biocredit cohorts

	Sure-Status	Biocredit	All
Total	372	232	604
Age (mean (min–max), IQR)	43 (18–81), 33–53	43 (18–78), 33–51	43 (18–81), 33–52
Gender (%F (n/N))	57% (211/372)	59% (137/232)	58% (348/602)
Triple vaccinated (n, %)	NA*	143 (61.6%)	143 (23.8%)
Double vaccinated (n, %)	314 (84.4%)	74 (40.0%)	388 (64.4%)
Partially vaccinated (n, %)	29 (7.8%)	4 (1.7%)	33 (5.5%)
Not vaccinated (n, %)	27 (7.3%)	10 (4.3%)	37 (6.2%)
Vaccination not disclosed (n, %)	2 (0.5%)	1 (0.3%)	3 (0.5%)
Days to symptom onset (median (IQR), N)	2 (1–3), 371	2 (1–3), 232	2 (1–3), 601
Days 0–3 (n, %)	304, 81.7%	186, 80.2%	490, 81.1%
Days 4–7 (n, %)	56, 15.1%	41, 17.7%	97, 16.1%
Days 8+ (n, %)	10, 2.7%	5, 2.2%	15, 2.5%
RT-qPCR SARS-CoV-2 Positivity (% (n/N))	31.7% (118/372)	53.7% (122/227)	40.1% (240/599)
Symptom (total n (%), in RT-qPCR positive n (%))			
Cough	248 (66.7%), 73 (61.3%)	139 (60.0%), 71 (58.2%)	387 (64.3%), 144 (60.0%)
Sore throat	129 (34.7%), 34 (28.6%)	103 (44.4%), 56 (45.9%)	232 (38.5%), 90 (37.4%)
Headache	123 (33.1%), 57 (47.9%)	80 (34.5%), 45 (36.9%)	203 (33.7%), 102 (42.3%)
Fever	106 (28.5%), 30 (25.2%)	54 (23.3%), 28 (22.9%)	160 (26.6%), 58 (24.1%)
Body aches	41 (11.0%), 21 (17.7%)	39 (16.8%), 29 (23.8%)	80 (13.3%), 51 (21.2%)
Runny nose	39 (13.2%), 20 (16.8%)	41 (17.7%), 31 (25.4%)	80 (13.3%), 51 (21.2%)
Loss taste	48 (12.9%), 19 (16.0%)	19 (8.2%), 10 (8.2%)	67 (11.1%), 29 (12.0%)
Loss smell	29 (7.8%), 9 (7.6%)	14 (6.0%), 7 (5.7%)	43 (7.1%), 16 (6.6%)
Chest pain	18 (4.8%), 7 (5.9%)	12 (5.2%), 8 (6.6%)	30 (5.0%), 15 (6.2%)
Fatigue	13 (3.5%), 4 (3.4%)	17 (7.3%), 10 (8.2%)	30, (5.0%), 14 (5.8%)
Shortness of breath/tight chest	13 (3.5%), 3 (2.5%)	9 (3.9%), 5 (4.1%)	22 (3.6%), 15 (6.2%)
Vomiting	11 (3.0%), 5 (4.2%)	2 (8.6%), 2 (1.6%)	13 (2.2%), 7 (2.9%)
Diarrhoea	9 (2.4%), 3 (2.5%)	3 (13%), 3 (2.5%)	12 (2.0%), 6 (2.5%)
Abdominal pain	6 (1.6%), 3 (2.5%)	1 (0.4%), 1 (0.8%)	7 (1.2%), 4 (1.7%)
Rash	3 (0.8%), 0 (0.0%)	1 (0.4%), 1 (0.8%)	4 (0.6%), 1 (0.4%)
Confusion	1 (0.3%), 0 (0.0%)	0 (0.0%)	1 (0.2%), 0 (0.0%)
Other	159 (42.7%), 68 (57.4%)	134 (57.8%), 85 (69.7%)	293 (48.7%), 153 (63.5%)

* Participants were enrolled before booster was rolled out in the UK.

NAnot availableRT-qPCRreverse transcription quantitative PCR

Overall, 240 participants (40.1%, 95% CI 36.1–44.1) were SARS-CoV-2 positive by RT-qPCR, 5 had indeterminate RT-qPCR results and the remaining were negative. Participants with indeterminate RT-qPCR results were excluded from further analysis.

RT-qPCR positivity was significantly higher (p<0.05) among the participants enrolled for the Biocredit evaluation cohort (53.7%, 95% CI 47–60.4) during December 2021 and March 2022 which coincided with the Omicron wave in the UK,^16^ than among the participants enrolled between August and October 2021 (31.7%, 95% CI 27.0– 36.7) when Delta was the dominant SARS-CoV-2 variant.

### Diagnostic evaluations

#### Sure-Status

The sensitivity and specificity for the Sure-Status Ag-RDT compared with RT-qPCR were 83.9% (95% CI 76.0–90.0) and 98.8% (95% CI 96.6–99.8) using NP swabs and 85.6% (95% CI 77.1–91.4) and 99.2% (95% CI 97.1–99.9) using AN swabs. For individuals with Cts<25, the sensitivity was 92.8% (95% CI 85.7–97.1) and 94.9% (95% CI 88.4–98.3) for NP and AN-swabs, respectively. Seven Ag-RDTs gave invalid results, one NP swab (0.03%) sample and six AN swab samples (1.6%). The difference in invalid results by sample type was not statistically significant by Fisher’s exact test (p=0.06381). Invalid Ag-RDT results were excluded from further analysis. Four SARS-CoV-2 positive cases were detected by NP only (3.4%) and six cases were detected by AN only (5.0%) but this discrepancy on sensitivity between swab types was not significant (p=0.43). The percentage of agreement of NP and AN swab using Sure-Status was 96.7% (95% CI 94.7 to 98.5) and inter-rater reliability was almost perfect (κ=0.918). Inter-rater reliability was strong for both NP (κ=0.871) and AN (κ=0.852) swabs when compared with RT-qPCR.

#### Biocredit

For the Biocredit Ag-RDT, the sensitivity and specificity were 81.2% (95% CI 73.1–87.7) and 99.0% (95% CI 94.7–86.5) with NP swabs and 79.5% (95% CI 71.3–86.3) and 100% (95% CI 96.5–100) with AN sampling compared with RT-qPCR. Sensitivity was 92.2% (95% CI 84.6–96.8) and 95.5% (95% CI 89.0–98.8) using NP and AN swabs among participants with Ct<25. Ten SARS-CoV-2 positive cases were detected solely by NP (8.2%) and 8 cases were detected only by AN (6.6%) but no significance on sensitivity was observed between NP and AN swabs for this brand of Ag-RDTs either (p=0.43). No invalid results were observed for this Ag-RDT. The percentage of agreement of NP and AN swab for Biocredit was 91.6% (95% CI 87.2–94.9) and inter-rater reliability was strong (κ=0.833). Inter-rater reliability was moderate for both NP (κ=0.790) and AN (κ=0.782) sampling compared with RT-qPCR. Diagnostic accuracy for both Sure-Status and Biocredit is displayed in table 2.

**Table 2:** Clinical sensitivity and specificity of Sure-Status and Biocredit using NP and Nasal Swab

All Ct values	TP	FP	TN	FN	Sensitivity	Specificity	NPV	PPV
Sure-Status								
NP swab	99	3	250	19	83.9% (76.0–90.0)	98.8% (96.6–99.8)	92.9% (89.7–95.2)	97.1% (91.4–99.0)
AN swab	101	2	246	17	85.6% (77.1–91.4)	99.2% (97.1–99.9)	93.5% (90.3–95.1)	98.1% (92.7–99.5)
Biocredit								
NP swab	99	1	104	23	81.2% (73.1–87.7)	99.0% (94.7–86.5)	81.9% (73.9–85.2)	99.0% (93.4–99.9)
AN swab	97	0	105	25	79.5% (71.3–86.3)	100% (96.5–100)	80.8% (74.8–85.6)	100% (100–100)
All NP	198	4	354	42	82.5% (77.1–87.1)	98.9% (97.2–99.7)	89.4% (86.5–91.7)	98.0% (94.9–99.2)
All AN	198	2	351	42	82.5% (77.1–87.1)	99.4% (97.9–99.9)	89.3% (86.4–91.7)	99.0% (96.1–99.8)

Sensitivity, specificity, NPV and PPV are shown with 95% CI.

ANanterior naresFNfalse negativesFPfalse positivesNPnasopharyngealNPVnegative predictive valuePPVpositive predicted valueTNtrue negativesTPtrue positives;

#### Head-to-head comparison of Sure-Status and Biocredit

We report non-significant difference in the diagnostic accuracy among participants with symptoms, irrespective of days since onset or vaccination status for all Ag-RDTs and swabbing combinations (all p>0.05). Both Biocredit and Sure-Status Ag-RDTs using both swab types had better sensitivities on detecting SARS-CoV-2 antigens in individuals with Ct values <25 than >30 (p=0.029 in NP and p=0.032 in AN for Sure-Status and p=0.018 and p=0.0002 for Biocredit).

**Figure 1:**
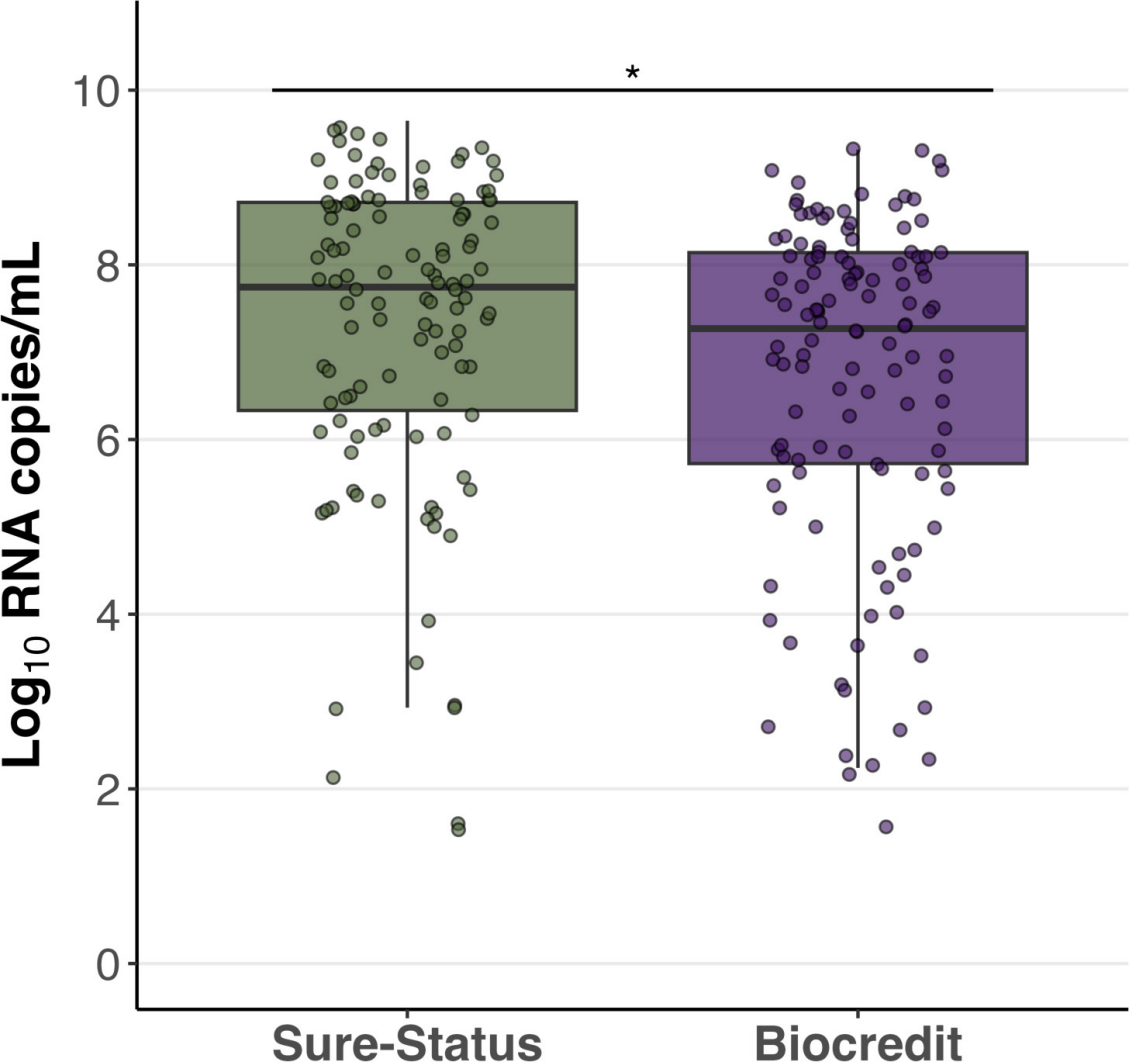
Boxplot of the SARS-CoV-2 viral load distribution of the RT-qPCR NP swabs used as reference standard for the participants enrolled for Sure-Status and Biocredit Ag-RDT evaluation. The whiskers show the maximum and minimum values and the vertical line the median. Asterisks indicate statistical significance between AN and NP swab types. AN, anterior nares; NP, nasopharyngeal; RDT, rapid diagnostic test; RT-qPCR, reverse transcription quantitative PCR.

**Figure 2:**
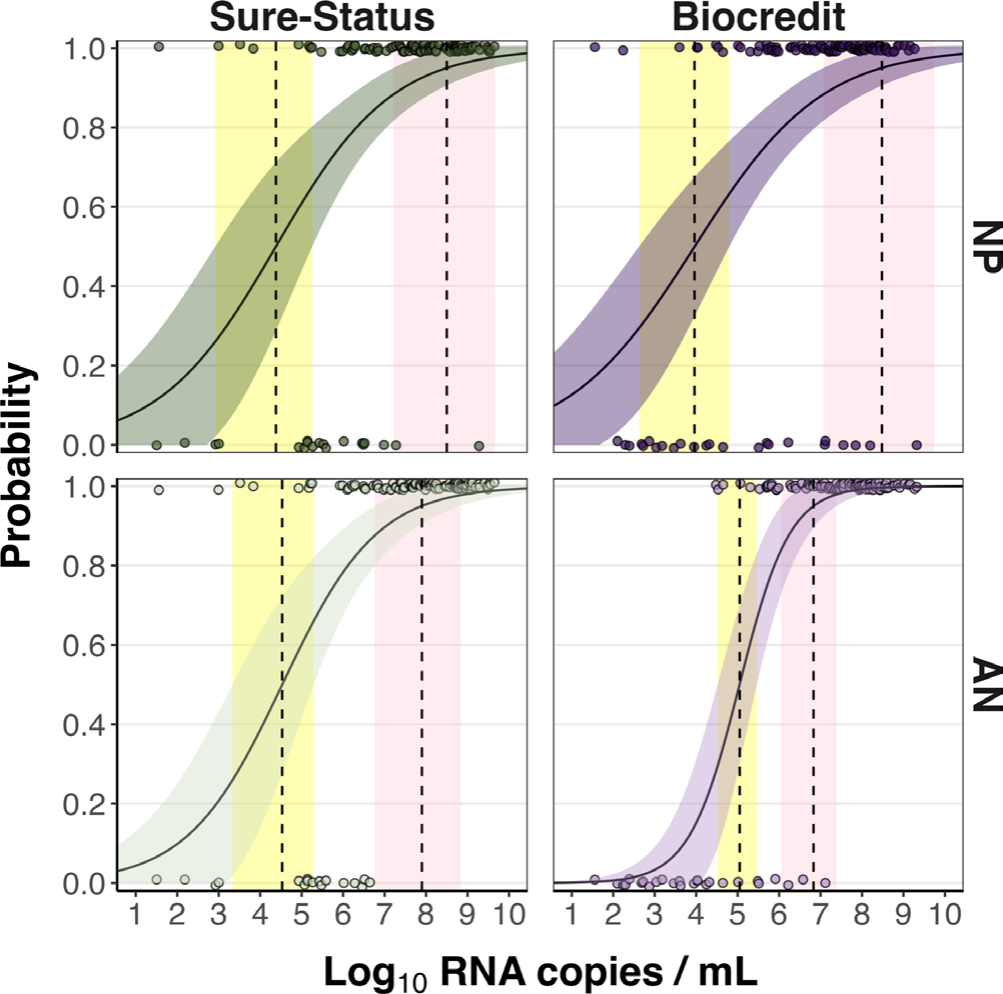
Limit of detection analyses of upper-respiratory samples positive by RT-qPCR for Sure-Status and Biocredit using AN and NP swabs. The log10 RNA copies on the x-axis were plotted against a positive (1.0) or negative (0.0) Ag-RDT result on the y-axis. Green (Sure-Status) and purple (Biocredit) curves show logistic regressions of the viral load on the Ag-RDT result; vertical dashed lines indicate log10 RNA copies subjected to the test at which 50% and 95% LoD of the samples are expected positive based on the regression results. No significant differences were observed. AN, anterior nares; LoD, limits of detection; NP, nasopharyngeal; RDT, rapid diagnostic test; RT-qPCR, reverse transcription quantitative PCR.

The RNA copy numbers per mL (RNA copies/mL) of RT-PCR NP swabs were calculated, and statistically higher viral loads were obtained for the Sure-Status cohort than Biocredit (figure 1) measured by Kruskal-Wallis (p=0.006). We determined the 50% and 95% limits of detection (LoD) for both Ag-RDTs and swab types based on a logistic regression model (figure 2). For Sure-Status, the RNA copies/mL for 50% LoD (LoD50) and 95% LoD (LoD95) were 2.4×10^4^ and 3.2×10^8^ for NP specimens and 3.4×10^4^ and 7.94×10^7^ for AN swabs. All participants who had a negative Ag-RDT result using AN swab and a positive result using NP swab had a viral load below LoD95 of both swab types (3.0×10^5^–4.4x10^6^ copies/mL). Five out of six participants who had a negative Ag-RDT result using NP swab and a positive result using AN swab had a viral load below LoD95 of both swab types (3.3×10^5^–1.8×10^7^ copies/mL) and one above (1.9×10^9^).

For Biocredit, the RNA copies/mL for LoD50 and LoD95 were 9.12×10^3^ and 3.02×10^8^ for NP specimens and 1.12×10^5^ and 6.7×10^6^ for AN swabs. Although the LoD95 was better for AN swabs for both Ag-RDT brands, there was no statistical difference in LoDs either by swab type or Ag-RDT brand (all p>0.05).

All participants who had a negative Ag-RDT result using AN swab and a positive result using NP swab had a viral load below LoD95 of both swab types (3.6×10^1^–3.7×10^6^ copies/mL). Seven out of eight participants who had a negative Ag-RDT result using NP swab and a positive result using AN swab had a viral load below LoD95 for NP swab (4.4x10^4^–1.6x10^8^ copies/mL) and one above (2.0×10^9^ copies/mL).

#### Quantitative read-out analysis

**Figure 3:**
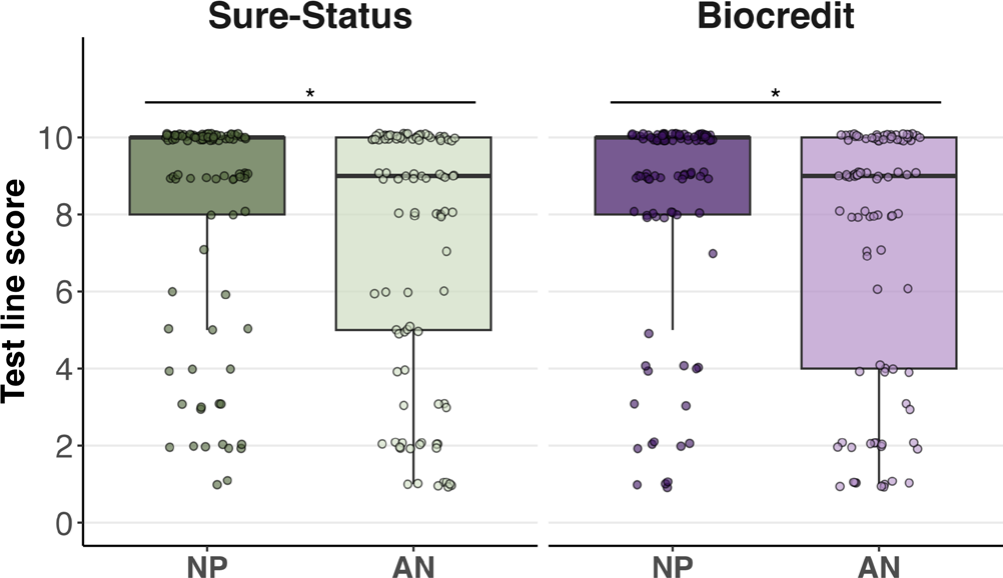
Boxplot of the scores of the test lines for both Ag-RDTs, Sure-Status and Biocredit, using AN and NP swabs. The whiskers show the maximum and minimum values and the vertical line the median. Asterisks indicate statistical significance between AN and NP swab types. AN, anterior nares; NP, nasopharyngeal; RDT, rapid diagnostic test.

Quantitative read-out in paired positive AN and NP swabs was more often higher for the NP swabs (40 instances higher on NP and four higher on AN in Sure-Status; and 35 instances higher on NP and 12 higher on AN in Biocredit) and gave significantly higher scores for both Ag-RDTs, Sure-Status (p=0.007) and Biocredit (p=0.013) (figure 3) measured by Kruskal-Wallis. Additionally, test line scores were analysed by RNA copies/mL and these had a positive correlation. For Biocredit, strong correlation using AN swabs (r_P_=0.727) but moderate using NP swabs (r_P_=0.591). For Sure-Status, both swab types had a moderate correlation to viral loads (NP swab r_P_=0.614 and AN swab r_P_=0.661).

## Discussion

To our knowledge, this is the first diagnostic clinical evaluation of Sure-Status Ag-RDT at the time of this publication, and the point estimates for sensitivity (≥80%) and specificity (≥97%) have shown satisfactory performance for both AN and NP swabs fulfilling the TPP WHO standards,^7^ although the lower bound of the 95% CI was below the TPP threshold. Further evaluations should be performed with a larger sample size for a more precise estimate.

For Biocredit Ag-RDT, there are five studies to date that have evaluated the performance of NP swabs reporting varied sensitivities from 52% to 85%.^4^ In this study, we reported a sensitivity and specificity of 81.2% (95% CI 73.1–87.7) and 99.0% (95% CI 94.7–86.5) fulfilling the WHO standards using NP swab when using the point estimates for sensitivity and specificity. Biocredit Ag-RDT point estimate for sensitivity was below the threshold (79.5%, 95% CI 71.3–86.3) of the WHO TPP when using AN. Differences in sensitivity between sample type and Ag-RDT brands were not statistically significant.

It was observed during Ag-RDT testing that AN swabs had higher viscosity when compared with NP swabs. Although not significant, this viscosity caused inappropriate sample flow in Sure-Status RDT, giving the higher invalid rate compared with NP swabs.

Even though the Ag-RDTs were evaluated in different cohorts (different recruitment times, SARS-CoV-2 variant, etc), results presented here demonstrate that AN swabs are equivalent to NP swabs for SARS-CoV-2 Ag-RDT testing, giving comparable sensitivities, LoD50 and LoD95 for both Ag-RDT brands evaluated here. Our results support previous findings where AN and NP swabs were compared for the Ag-RDT Standard-Q (SD Biosensor, Korea) in Lesotho^5^ and also obtained lower sensitivities in AN (67.3%) than NP (70.2%) swabs.^5^ Studies on RT-qPCR have found lower sensitivity using AN swabs compared with NP swabs consistently.^9^ However, the difference in sensitivity was only significant for patients with viral loads <10^3^ copies/mL,^17^ and this threshold is not relevant to Ag-RDTs of which the LoD ranges between 10^4^ and 10^8^ RNA copies/mL in swabs.^17^ ^11 18^

Quantitative assessment of the test line scores showed that test line intensity was significantly higher on NP swabs than AN swabs. The line intensity is an important component of home testing as studies have shown fainter lines are more difficult to interpret for a layperson, likely due to lower signal intensity.^19^ In a user experience home-based study, 77.1% of the cases that the participants interpreted wrongly as negative being positive were weak and moderate positives, while only 22.9% were strong positives.^19^ The lower intensity of the AN swab compared with NP swab is likely attributed to the differences in SARS-CoV-2 viral loads in the respiratory tract. Studies have found lower viral loads on AN swabs compared with NP swabs.^17^ Statistical analysis supported this hypothesis, where a positive correlation between viral loads and Ag-RDT test line scores was shown. Further implementation studies on Ag-RDT test results interpretation by patients or within a home testing setting are urgently needed to drive self-testing to scale.

This study has several strengths, the use of standardised sampling methods, independent blinded readers, robust statistical analysis, quantitative assessment of Ag-RDT test line results and the evaluation of two approved WHO-EUL Ag-RDT test brands, qualifying it to have high global public health relevance.^20^

The main limitation of this study is that, although the operators were blinded to each other and to the SARS-CoV-2 RT-qPCR result, they were not blinded to the swab type as the shape and size of these are different and the tests were done in parallel. We do not expect that this could have caused a bias in reading the Ag-RDT results as the reference standard result was unknown for the operators and data was QC, but this is a consideration for future studies. Another limitation of this study is that the AN swabs were always taken last. The order of sample collection could have negatively biased the results obtained for AN swabs caused by possible sample depletion. However, in the two studies that compared Ag-RDT using AN swabs, the AN swab was collected first, and in both studies, the sensitivity obtained with AN swabs was lower than when using NP swabs.^5 6^ Further, studies on RT-qPCR found also lower sensitivity using AN swabs compared with NP swabs,^9^ even when AN swabs were collected first.^17 21 22^ Thereby it is unlikely that the order of the swabs impacted sample availability for AN and NP sampling.

An interesting point to discuss is the circulation of different SARS-CoV-2 variants of concern during the study period. Sure-Status was evaluated during the Delta wave and Biocredit during the Omicron wave. A later study to this one evaluated the analytical sensitivity of Biocredit and Sure-Status using clinical samples positive for Omicron and Delta.^11^ Interestingly, both Ag-RDT brands had higher sensitivity point estimates among Omicron positive samples than Delta; however, this was not significant, suggesting that it is unlikely that the SARS-CoV-2 strain present at the time of evaluation had an impact on the sensitivity estimates of the Ag-RDTs.

In conclusion, this study demonstrates the sensitivity of two SARS-CoV-2 Ag-RDTs using AN-sampling is comparable to that of NP-sampling. AN-sampling can be performed with less training, reduces patient discomfort, and enables scaling up of antigen testing strategies. Test line intensity, however, is lower when using AN swabs, which could negatively influence the interpretation of the Ag-RDT results. Additional studies on Ag-RDTs using AN swabs on self-interpretation by a layperson are needed, and further education around how to interpret a positive Ag-RDT to the wider community.

## supplementary material

10.1136/bmjresp-2023-001747 online supplemental material 1

## Data Availability

Data are available on reasonable request.
