# Supplementary material for: Head-to-head comparison of anterior nares and nasopharyngeal swabs for SARS-CoV-2 antigen detection in a community drive-through test centre in the UK
Source: BMJ Open Respir Res. 2025 Mar 22;12(1):e001747. doi: 10.1136/bmjresp-2023-001747 (PMC11934397; doi:10.1136/bmjresp-2023-001747)
Supplement: online supplemental material 1 [file bmjresp-12-1-s001.pdf]

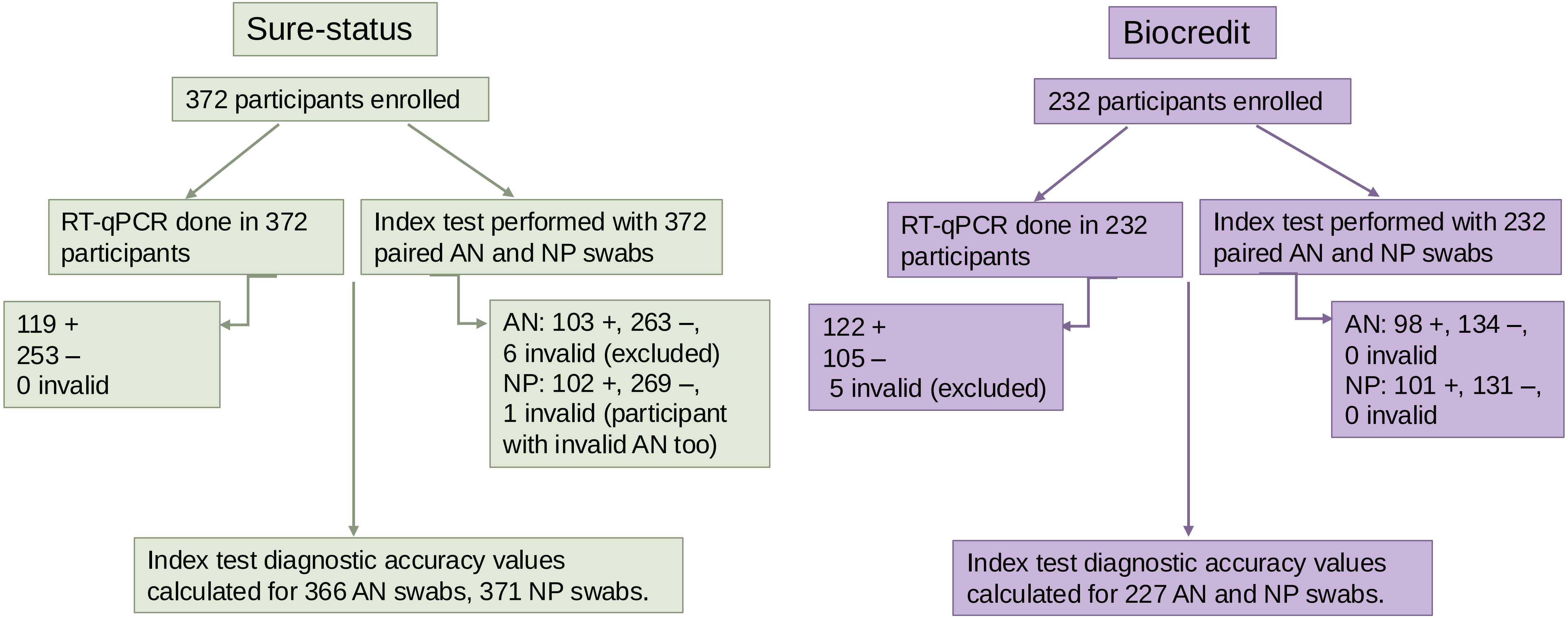
Sure-status
372 participants enrolled
RT-qPCR done in 372 participants
Index test performed with 372 paired AN and NP swabs
119 +
253 –
0 invalid
AN: 103 +, 263 –,
6 invalid (excluded)
NP: 102 +, 269 –,
1 invalid (participant with invalid AN too)
Index test diagnostic accuracy values calculated for 366 AN swabs, 371 NP swabs.
Biocredit
232 participants enrolled
RT-qPCR done in 232 participants
Index test performed with 232 paired AN and NP swabs
122 +
105 –
5 invalid (excluded)
AN: 98 +, 134 –,
0 invalid
NP: 101 +, 131 –,
0 invalid
Index test diagnostic accuracy values calculated for 227 AN and NP swabs.
